# Bariatric Surgery Impacts Retinal Vessel Status Assessed by Optical Coherence Tomography Angiography: A Prospective 12 Months Study

**DOI:** 10.3390/jcm14248644

**Published:** 2025-12-05

**Authors:** Xavier Carreras-Castañer, Sofía Batlle-Ferrando, Rubén Martín-Pinardel, Teresa Hernández, Cristian Oliva, Irene Vila, Rafael Castro-Dominguez, Andrea Mendez-Mourelle, Alfredo Adán, Diana Tundidor, Ana de Hollanda, Emilio Ortega, Amanda Jiménez, Javier Zarranz-Ventura

**Affiliations:** 1 Department of Ophthalmology, Hospital Clínic of Barcelona, 08036 Barcelona, Spain; drxcarreras@gmail.com (X.C.-C.); batlle@clinic.cat (S.B.-F.); tessa.hrndz@gmail.com (T.H.); coliva@clinic.cat (C.O.); irvila@clinic.cat (I.V.); rcastrod@recerca.clinic.cat (R.C.-D.); anmendez@clinic.cat (A.M.-M.); amadan@clinic.cat (A.A.); 2 August Pi i Sunyer Biomedical Research Institute (IDIBAPS), 08036 Barcelona, Spain; rbnmartinpinardel@gmail.com (R.M.-P.); dtundidor@santpau.cat (D.T.); amdehol@clinic.cat (A.d.H.); eortega1@clinic.cat (E.O.); ajimene1@clinic.cat (A.J.); 3 Department of Surgery and Medical-Surgical Specialties, Faculty of Medicine and Health Sciences, Universitat de Barcelona, 08036 Barcelona, Spain; 4 Department of Endocrinology, Hospital Clínic of Barcelona, 08036 Barcelona, Spain; 5 Centro de Investigación Biomédica en Red de la Fisiopatología de la Obesidad y Nutrición (CIBEROBN), 08036 Barcelona, Spain

**Keywords:** microvascular changes, macular perfusion, macular vessel density, optical coherence tomography angiography, optic nerve, bariatric surgery, obesity, oculomics, body mass index, retina

## Abstract

**Objectives:** To assess retinal microvascular changes in patients with Grade II and III obesity before and after bariatric surgery using Optical Coherence Tomography Angiography (OCTA), and to compare these metrics with age- and sex-matched healthy controls. **Methods:** Prospective, consecutive, longitudinal cohort study with a 12-month follow-up. Grade II and III obese patients scheduled for bariatric surgery underwent comprehensive ophthalmic examinations, including OCTA imaging, prior to the surgery and postoperatively at 1 month, 6 months, and 12 months post-surgery. **Results:** A total of 43 eyes from 43 patients with obesity (one eye per patient) were included at baseline. At 12 months post-surgery, there was a significant increase in vessel density (VD) (16.70 vs. 17.68; *p* < 0.01) and perfusion density (PD) (0.406 vs. 0.433; *p* < 0.01), reaching values comparable to those of the control group (17.73 and 0.434, respectively). Significant reductions were also observed in body mass index (BMI) (43.74 vs. 29.53; *p* < 0.01), body weight (122.44 kg vs. 81.90 kg; *p* < 0.01), and intraocular pressure (IOP) (15.72 mmHg vs. 14.16 mmHg; *p* < 0.01). **Conclusions:** This study demonstrates a compelling association between obesity and retinal microvascular impairment, highlighting the efficacy of bariatric surgery not only in achieving substantial weight loss but also in improving the retinal perfusion of these patients, achieving metrics at 12 months comparable to age- and sex-matched healthy controls at baseline. These findings raise the hypothesis of the potential utility of OCTA as a monitoring tool for tracking the microvascular status in patients with obesity undergoing bariatric surgery in a longitudinal manner.

## 1. Introduction

Obesity is a multifactorial metabolic disorder that has become a major global public health challenge, contributing to substantial morbidity and mortality across all age groups, including children and adolescents. The World Health Organization (WHO) identifies obesity as the most prevalent chronic condition affecting adults worldwide [1]. It is defined by an excessive accumulation of adipose tissue, commonly assessed using the Body Mass Index (BMI), which is calculated by dividing body weight (in kilograms) by the square of height (in meters). In adults, obesity is classified into three grades: Grade I (BMI > 30 kg/m^2^), Grade II (BMI > 35 kg/m^2^), and Grade III (BMI > 40 kg/m^2^) [2].

Obesity is strongly associated with chronic inflammation, primarily driven by the dysregulated production of pro-inflammatory cytokines that negatively affect vascular function [3]. Recent studies have identified elevated levels of intravascular superoxide in individuals with obesity, which reduces the bioavailability of nitric oxide, a key mediator of endothelial function, thereby contributing to endothelial dysfunction [4]. Small vessel disease (SVD) encompasses a group of conditions characterized by structural and functional damage to arterioles and capillaries, leading to impaired or disrupted blood flow in affected tissues. SVD predominantly impacts organs with high perfusion demands, such as the brain, kidneys, and retina [5].

The relationship between obesity, weight loss, and ocular pathology has been extensively investigated [6,7]. Recent evidence suggests that bariatric procedures, such as laparoscopic sleeve gastrectomy, can significantly enhance microvascular function in individuals with severe obesity [8]. Notably, one study reported a significant increase in retinal thickness following bariatric surgery, independent of diabetic status [9]. Similarly, Dogan et al. observed increases in central macular thickness (CRT) and total macular volume (MV) in post-surgical patients [10]. Gonul et al. further demonstrated a direct correlation between choroidal thickness and BMI, with progressive choroidal thinning observed after weight loss surgery [11]. These findings underscore the role of CRT as a measurable structural biomarker of retinal response to weight reduction interventions [12]. Regarding the retinal nerve fiber layer (RNFL), studies have shown that individuals with obesity tend to exhibit reduced RNFL thickness compared to non-obese controls, potentially serving as an indicator of neurodegeneration [13]. Conversely, Elshazly et al. reported a significant decrease in intraocular pressure (IOP) three months after bariatric surgery, although no corresponding changes were observed in RNFL or ganglion cell complex (GCC) thickness [12].

Optical Coherence Tomography Angiography (OCTA) has emerged as a robust imaging modality for the objective and quantitative evaluation of retinal microvasculature. Its ability to detect subtle vascular alterations has enabled the identification of clinically relevant associations with systemic parameters and dysfunctions in other organ systems [14,15]. The association between obesity, bariatric surgery, and retinal microvascular changes assessed by OCTA has been investigated, although findings remain limited and somewhat inconsistent. Agarwal et al. reported elevated choroidal vascularity index and retinal capillary density index in obese individuals compared to healthy controls but found no significant changes following three months of bariatric surgery, exercise, and dietary interventions [16]. In contrast, Laiginhas et al. observed increased vascular density in the parafoveal deep vascular plexus and a reduction in the perimeter of the foveal avascular zone post-surgery, suggesting a potential improvement in retinal microcirculation [17].

This prospective study aims to evaluate the impact of bariatric surgery and the resulting weight loss on the retinal microvascular status, as assessed by Optical Coherence Tomography Angiography (OCTA) over a 12-month period. Retinal vascular metrics obtained from operated patients will be compared to those of a control group of non-obese individuals matched by age and sex. Comparisons will be made at baseline and across all postoperative timepoints, providing a reference framework to assess longitudinal changes in retinal microcirculation.

## 2. Methods

### 2.1. Study Design and Ethics

Prospective, consecutive, longitudinal 12-month follow-up cohort study. Patients with Grade II and Grade III obesity were recruited from the Bariatric Surgery Unit and referred to the Ophthalmology department prior to the surgery. All participants received a comprehensive ophthalmic evaluation, including a full suite of retinal imaging tests using OCTA, alongside the collection of ocular and systemic clinical data. Ethical approval was obtained from the Institutional Review Board of Hospital Clínic de Barcelona (study reference HCB/2021/0622). Written informed consent was obtained from all participants. The study was conducted in accordance with the tenets of the Declaration of Helsinki.

### 2.2. Inclusion and Exclusion Criteria

Patients with Grade II and Grade III obesity scheduled for bariatric surgery were invited to participate in the study. Those who consented and met the inclusion criteria underwent a comprehensive ophthalmic evaluation in the Ophthalmology Department. Exclusion criteria included the presence of ocular comorbidities (e.g., macular edema, history of ocular surgery, macular laser treatment, intravitreal therapy), media opacities, or inability to complete the full ophthalmic examination or provide written informed consent. The control group consisted of healthy volunteers recruited through social media campaigns coordinated by the hospital’s communications department.

### 2.3. Ocular and Systemic Data

Ocular data collected included best-corrected visual acuity (BCVA), slit-lamp direct biomicroscopy, intraocular pressure measurement, retinal fundus examination, and ocular biometry (IOL Master 500, Carl Zeiss Meditec, Dublin, CA, USA). BMI was calculated based on each patient’s measured weight and height. A comprehensive set of retinal imaging tests, including OCT and OCTA, was performed as detailed below. Systemic data collected included demographic and clinical variables such as age, sex, smoking status, systolic and diastolic blood pressure, presence of arterial hypertension, and diabetes mellitus.

### 2.4. Structural Optical Coherence Tomography (OCT) and OCTA Imaging Protocols

All OCT and OCTA images were acquired using the Cirrus HD-OCT system (Carl Zeiss Meditec, Dublin, CA, USA). Structural OCT scans were performed using the 6 × 6 mm Macular Cube 512 × 128 protocol, while OCTA scans employed 6 × 6 mm cube acquisitions centered on the fovea. Image quality was rigorously assessed, and scans with artifacts, segmentation errors, or a signal strength index (SSI) below 7 were excluded from analysis. Structural OCT parameters included CRT, MV, and average macular thickness (AMT). OCTA metrics were quantified using the AngioPlex Metrix software (version 11.0.0) (Carl Zeiss Meditec, Dublin, CA, USA), focusing on the superficial capillary plexus, defined by the boundaries between the internal limiting membrane and the inner plexiform layer. OCTA measurements included vessel density (VD), perfusion density (PD, 0–1), foveal avascular zone area (FAZa, mm^2^), perimeter (FAZp, mm), and circularity (FAZc, %). No manual adjustments were made to the segmentation slabs.

### 2.5. Statistical Analysis

Quantitative variables were summarized using mean, standard deviation (SD), and standard error of the mean (SEM), while qualitative variables were described using absolute frequencies and percentages. The normality of data distributions was assessed using the Shapiro–Wilk test, and homogeneity of variances was evaluated with Levene’s test. Independent group comparisons were performed using ANOVA, Kruskal–Wallis, and Chi-square tests, as appropriate. For pairwise comparisons, Welch’s t-test and the Mann–Whitney U test were applied. Linear mixed-effects models were used to make pairwise group comparisons while taking into account repeated measures. No correction for multiple testing was performed. A *p*-value < 0.05 was considered statistically significant. All statistical analyses were conducted in R (v4.1.2) using RStudio (version 22021.09.1 + 372) and the package nlme (version 3.1.153) for mixed-effects models.

## 3. Results

### 3.1. Study Cohort

**Figure 1:**
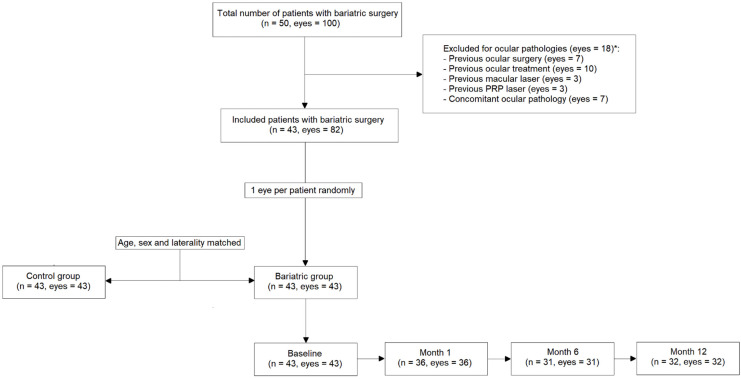
Consolidated standard of reporting trials (CONSORT)-style flow diagram describing included and excluded patients and eyes. (*= non exclusive categories, the same eye can present >1 condition).

Figure 1 presents a CONSORT-style flow diagram detailing the inclusion and exclusion process for study eyes based on predefined eligibility criteria. To mitigate potential bilaterality bias, only one eye per participant was randomly selected and included, yielding a final sample of 43 eyes (one eye per patient; *n* = 43). The control cohort comprised 43 eyes from healthy individuals, matched for age, sex, and laterality. The number of OCTA scans excluded due to low quality (SSI < 7) at baseline was 3, at 1 month 3, at 6 months 1, and at 12 months 1, and in controls it was 2.

### 3.2. Baseline Demographic and Clinical Characteristics of Study Groups

Table 1 summarizes the baseline characteristics of the study population, comparing the group with obesity (*n* = 43) with healthy controls (*n* = 43). Significant differences were observed in body weight (122.44 kg vs. 68.35 kg) and body mass index (BMI: 43.74 vs. 24.21), with both comparisons reaching statistical significance (*p* < 0.05). No statistically significant differences were found between groups regarding age, sex, or height. Table 2 details the cardiovascular clinical characteristics of study patients.

### 3.3. Baseline OCTA and Structural OCT Characteristics of Study Groups

Table 3 presents the baseline OCTA characteristics of the study population. Statistically significant differences were found between patients with obesity and healthy controls in VD (16.70 vs. 17.73; *p* < 0.05), PD (0.40 vs. 0.43; *p* < 0.05), and FAZc (0.65 vs. 0.74; *p* < 0.05). No significant differences were observed between groups in other FAZ metrics, structural macular OCT parameters, or optic nerve head (ONH) measurements.

### 3.4. Systemic and Ocular Characteristics Changes Following Bariatric Surgery: 12-Month Progression

**Figure 2:**
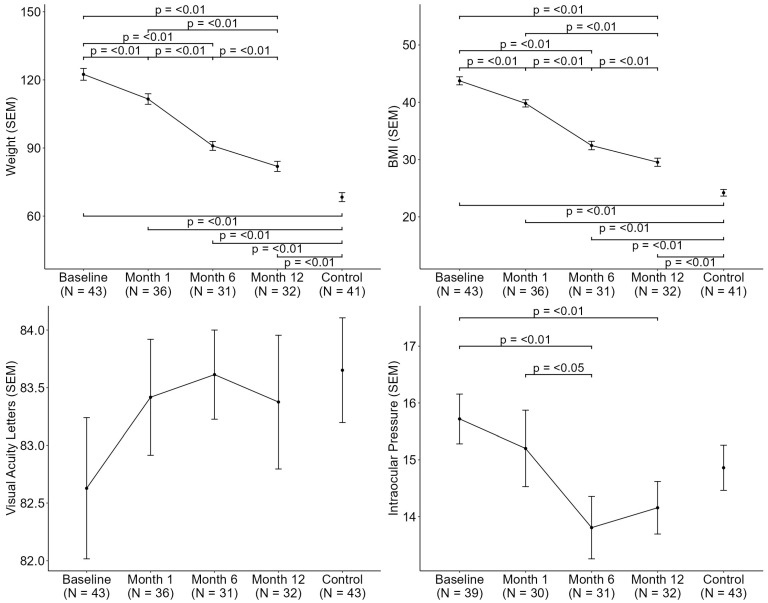
Systemic and ocular characteristics changes following bariatric surgery from baseline to month 12. (BMI: Body mass index; SEM: Standard error of the mean).

A significant and progressive reduction in anthropometric parameters was observed in the cohort with obesity over the 12-month period following bariatric surgery (Figure 2, top). Body weight decreased from 122.44 kg at baseline to 81.90 kg at 12 months (*p* < 0.01), accompanied by a corresponding reduction in BMI. Despite this improvement, the control group maintained significantly lower weight and BMI values at 12 months (81.90 kg vs. 68.35 kg; BMI: 29.53 vs. 24.21; both *p* < 0.01). Intraocular pressure (IOP) also showed a significant decline from baseline to month 6 (15.72 mmHg vs. 13.81 mmHg; *p* < 0.01), followed by stabilization through month 12 (Figure 2, bottom).

### 3.5. Longitudinal Optical Coherence Tomography Angiography Parameters Evolution over 12 Months Post-Bariatric Surgery

**Figure 3:**
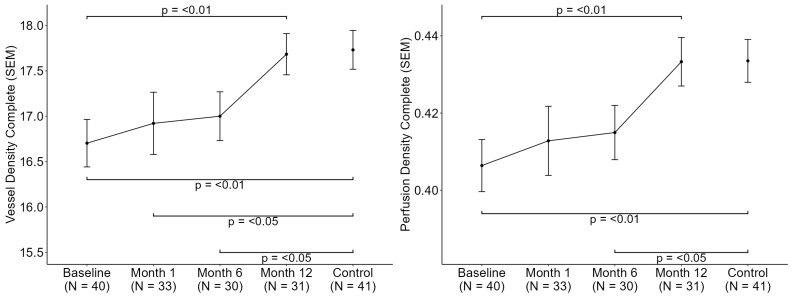
Longitudinal Assessment of Macular OCTA parameters over 12 Months (6 × 6 mm). Vessel density and perfusion density (SEM: Standard error of the mean).

**Figure 4:**
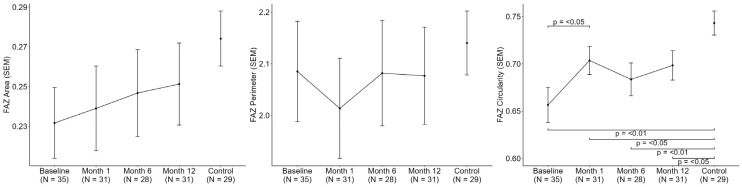
Longitudinal Assessment of Macular OCTA parameters over 12 Months (6 × 6 mm). Foveal avascular zone parameters (FAZ: Foveal avascular zone; SEM: Standard error of the mean).

Longitudinal analysis of OCTA parameters revealed a progressive improvement in retinal microvascular metrics following bariatric surgery, as shown in Figure 3 and Figure 4. Significant increases were observed in VD, PD, and FAZc over the 12-month follow-up period (*p* < 0.05). A sensitivity analysis adjusted by SSI was conducted and did not yield significant results. These findings suggest a potential reversal of obesity-associated microvascular alterations in the retina after substantial weight loss.

### 3.6. Structural Macular and Optic Nerve Head OCT Changes over 12 Months Post-Bariatric Surgery

**Figure 5:**
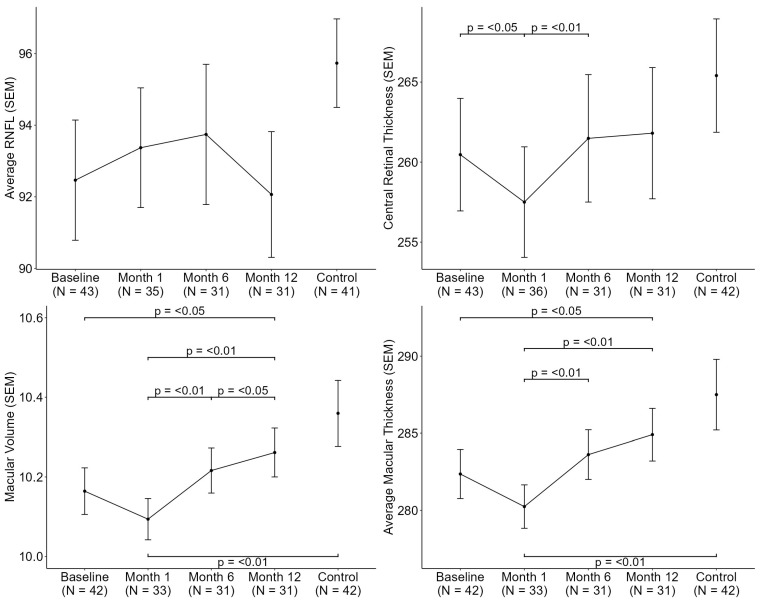
Longitudinal Progression of Structural Macular and Optic Nerve OCT Metrics. (RNFL: Retinal nerve fiber layer; SEM: Standard error of the mean).

As shown in Figure 5, a significant transient reduction in CRT was observed at month 1 (260.47 µm to 257.50 µm; *p* < 0.05), followed by a progressive increase through month 12. A similar trend was noted for macular volume and average macular thickness, both of which were significantly higher at 12 months compared to baseline (macular volume: 10.16 mm^3^ vs. 10.26 mm^3^; *p* < 0.05; average macular thickness: 282.36 µm vs. 284.90 µm; *p* < 0.05).

## 4. Discussion

This study demonstrates a clear association between obesity and retinal microvascular alterations, and further characterizes the progressive changes in retinal vascular parameters following bariatric surgery in patients without clinically evident retinal disease. At baseline, patients with obesity exhibited significantly reduced VD and PD compared to healthy controls. Longitudinal follow-up revealed notable improvements in these parameters over 12 months post-surgery, suggesting a potential reversal of obesity-induced microvascular compromise. These findings are clinically relevant, as the retinal microvascular changes observed may reflect broader systemic microvascular responses to bariatric surgery. Given the retina’s accessibility and sensitivity to systemic vascular alterations, OCTA could serve as a valuable surrogate marker for evaluating microvascular health in obese patients undergoing metabolic interventions.

OCTA has emerged as a pivotal tool for assessing retinal microcirculation. Its non-invasive, high-resolution imaging capabilities allow for detailed visualization of both retinal and choroidal vasculature, facilitating the study of systemic disease impacts on ocular structures. A comparative overview of previously published data is provided in Table 4. For instance, Dogan et al. [18] and Agarwal et al. [16] employed OCTA to demonstrate reduced VD and PD in individuals with obesity. Dogan et al. focused on optic disk and retinal vascular densities, reporting significant reductions in patients with obesity compared to controls. Similarly, Agarwal et al. documented decreased choroidal thickness and macular perfusion, offering structural evidence of obesity-related microvascular impairment. However, both studies were limited by their cross-sectional design, which precluded assessment of temporal changes or the impact of therapeutic interventions.

In the short term, studies such as those by Laiginhas et al. [17] and ElShazly et al. [12] have also employed OCTA to investigate microvascular changes following bariatric surgery. Laiginhas et al. reported improvements in systemic microvascular perfusion, suggesting a broader vascular benefit associated with weight loss. However, their study did not include specific macular perfusion metrics or a detailed analysis of retinal vascular density, limiting its applicability to ocular microcirculation. Conversely, ElShazly et al. utilized OCTA to assess optic nerve head perfusion and retinal nerve fiber layer (RNFL) thickness. While they observed reductions in intraocular pressure and enhancements in optic nerve perfusion, no significant changes were reported in retinal microvascular parameters or RNFL thickness after a three-month follow-up. These findings highlight the variability in OCTA outcomes depending on the anatomical region assessed and the relevance of the duration of follow-up.

Our study builds upon existing evidence by demonstrating not only significant improvements but also complete normalization of macular microvascular parameters at 12 months following bariatric surgery. Utilizing advanced OCTA metrics, we quantified changes in VD and PD across the superficial capillary plexus using 6 × 6 mm scan protocols. These findings underscore the reversibility of obesity-induced microvascular damage and highlight the importance of sustained weight loss in restoring retinal vascular integrity. Moreover, our longitudinal design addresses a critical gap left by previous cross-sectional or short-term studies. The normalization of macular microvascular parameters observed in our cohort carries important clinical implications. Retinal microcirculation serves as a readily accessible surrogate marker for systemic microvascular health. Improvements in macular vascular density and perfusion suggest that bariatric surgery may reverse early vascular damage associated with obesity, potentially mitigating the risk of vision-threatening conditions such as diabetic retinopathy or age-related macular degeneration. Furthermore, these results reinforce the utility of OCTA not only as a diagnostic modality but also as a valuable tool for monitoring therapeutic efficacy in patients undergoing metabolic interventions. In light of our data, OCTA may have the potential clinical utility to identify different responses after bariatric surgery in specific groups of patients, allowing a personalized systemic management in each particular case in the future.

Structural OCT analysis revealed a marginal increase in MV and CRT at 12 months postoperatively, following an initial transient reduction at month 1. These parameters subsequently recovered and closely approximated the values observed in the control group. These findings are consistent with previous studies [9,10,13], supporting the notion that baseline structural alterations are characteristic of individuals with obesity and may reflect systemic improvements in perfusion following bariatric surgery. Interestingly, this recovery pattern was not observed in RNFL thickness, which remained significantly thinner in the obese group compared to controls throughout the preoperative and 12-month postoperative assessments. This persistent thinning may indicate irreversible neurodegenerative changes associated with Grade II and III obesity, as previously suggested by Laiginhas et al. [13].

**Table 4:** Comparative Overview of Studies Assessing Retinal Changes Associated with Obesity and Weight Loss.

Author	Sample Size	Control Group	Follow-Up Duration	Study Design	Country	Parameters Evaluated	Methods Used	Main Results	Limitations
Agarwal et al. (2020) [16]	50	Yes (25 age and sex matched controls)	6 months	Prospective	India	Retinochoroidal structural alterations	OCT (No OCTA)	Significant improvement in retinal and choroidal thickness post-weight loss.	Limited to structural outcomes; functional changes not assessed.
ElShazly et al. (2022) [12]	45	No control group	3 months	Prospective	Egypt	IOP, RNFL thickness, optic nerve head blood flow	OCTA, RNFL	IOP reduction and improved optic nerve head perfusion; no RNFL changes.	Short follow-up period; no comparison group for validation.
Laiginhas et al. (2021) [17]	35	Yes (15 controls)	12 months	Prospective	Portugal	Microvascular perfusion changes	OCTA and systemic vascular assessments	Improved microvascular perfusion post-gastric bypass surgery.	Small sample size; patient demographics not diverse.
Dogan et al. (2023) [18]	60	Yes (30 controls)	No follow-up (cross-sectional)	Cross-sectional	Turkey	Optic disk and retinal vascular densities	OCTA	Reduced vascular density in obese patients compared to controls.	Cross-sectional design; no post-weight-loss evaluation.
Alacamli et al. (2023) [19]	80	No control group	6 months	Prospective	Romania	Microvascular changes in obesity	OCTA	Detection of early microvascular changes in obese patients.	No differentiation between obesity-related changes and comorbidities.
This study	43	Yes (43 age and sex matched controls)	12 months	Prospective	Spain	Functional, structural, and microvascular retinal perfusion changes	OCT and OCTA	Improved microvascular perfusion post-bariatric surgery, compared to the control group at month 12	-

Despite the strengths of our study—including its prospective design, relatively large sample size, and extended follow-up period—several limitations should be acknowledged. First, we experienced a 22% loss to follow-up, likely due to the high number of medical appointments patients must attend during the first postoperative year, including endocrinological, surgical, nutritional, and, in some cases, psychological consultations. It should be noted that OCTA images were captured in independent research visits on different dates in the ophthalmology department, which is placed in a geographically distinct location, adding complexity to the study and increasing the burden of the visits to patients. Second, the commercial version of the software used (Zeiss Angioplex, version 11.0.0, Carl Zeiss Meditec, Dublin, CA, USA) only provides OCTA metrics of the SCP and not the DCP, which may behave differently. The role of this plexus will be investigated in future studies using research software. Third, although the control group was matched for age and sex, it was not longitudinally monitored, which may have introduced bias in temporal comparisons. Fourth, our study population was restricted to individuals without advanced systemic or ocular comorbidities, potentially limiting the generalizability of our findings to more diverse populations. Additionally, the sample size was constrained by the annual volume of bariatric procedures performed at our center. Nonetheless, these limitations did not compromise our ability to detect statistically significant differences in most of the parameters assessed.

In conclusion, this study represents a pioneering investigation into retinal microvascular changes following bariatric surgery, with a 12-month longitudinal follow-up in a relatively large cohort of patients. While further validation in diverse populations is warranted, our findings consistently demonstrate the presence of subclinical microvascular impairment in obese individuals and highlight the efficacy of bariatric surgery in enhancing retinal perfusion. The observed improvements may reflect similar vascular changes occurring in other organ systems, underscoring the potential of OCTA as a non-invasive imaging modality for the objective assessment of microvascular health in systemic conditions. These results advocate for the integration of OCTA in longitudinal monitoring protocols, offering valuable insights into the progression and reversibility of obesity-related microvascular alterations.

## Figures and Tables

**Table 1:** Demographics and clinical characteristics of study eyes. *p* values: Basal-Month 1 (a); Baseline-Month 6 (b); Baseline-Month 12 (c); Baseline-Control (d). (BMI: Body mass index; logMAR: Logarithm of the minimum angle of resolution).

Characteristics, Mean (SD) (SEM)	Baseline	Month 1	Month 6	Month 12	Controls	*p*-Value
DEMOGRAPHICS						
Age	49.26 (10.52)(1.60)	-	-	-	49.28 (10.79)(1.65)	0.990
Sex, male, *n* (%)	16 (37.2%)	-	-	-	16 (37.2%)	1.000
Weight (kg)	122.44 (17.04)(2.60)	111.57 ^a^ (13.91)(2.32)	90.94 ^b^ (10.97)(1.97)	81.90 ^c^ (12.56)(2.22)	68.35 ^d^ (12.72)(1.99)	a,b,c,d
Height (m)	1.66 (0.09)(0.01)	-	-	-	1.68 (0.09)(0.01)	0.425
BMI (kg/m^2^)	43.74 (4.62)(0.70)	39.81 ^a^ (3.77)(0.63)	32.45 ^b^ (4.12)(0.74)	29.53 ^c^ (4.02)(0.71)	24.21 ^d^ (3.73)(0.58)	a,b,c,d
OCULAR DATA						
Visual Acuity (LogMAR)	82.63 (4.01)(0.61)	83.42 (3.02)(0.50)	83.61 (2.16)(0.39)	83.38 (3.28)(0.58)	83.65 (2.98)(0.45)	-
Spherical Equivalent	−0.65 (2.15)(0.34)	-	-	-	−0.13 (2.26)(0.35)	0.920
Axial Length	23.66 (1.25)(0.20)	-	-	-	23.74 (1.00)(0.15)	0.441
Intraocular Pressure	15.72 (2.73)(0.44)	15.20 (3.68)(0.67)	13.81 ^b^ (3.06)(0.55)	14.16 ^c^ (2.62)(0.46)	14.86 ^d^ (2.61)(0.40)	b,c,d
Eyes	43	36	31	32	43	

**Table 2:** Cardiovascular clinical characteristics of study patients. (BP: Blood pressure; CAI: Carbonic anhydrase inhibitors; HT: Hypertension; SD: Standard deviation; SEM: Standard error of the mean).

Baseline Cardiovascular Characteristics, Mean (SD)(SEM)	Patients with Obesity	Controls	*p*-Value
Systolic BP (mmHg)	131.69 (14.96)(2.49)	120.84 (15.29)(2.33)	<0.05
Dyastolic BP (mmHg)	82.47 (8.28)(1.38)	81.02 (9.59)(1.46)	0.584
Heart Rate	74.39 (12.31)(2.05)	70.86 (10.13)(1.55)	0.195
HbA1c	6.07 (1.14)(0.18)	5.41 (0.33)(0.05)	<0.05
Diabetes Mellitus	No: 20 (46.5%) Yes: 14 (32.6%) Pre-DM: 9 (20.9%)	-	
Cerebrovascular disease *n* (%)	0 (0.0%)	0 (0.0%)	1.000
Ischemic heart disease *n* (%)	0 (0.0%)	1 (2.3%)	1.000
Peripheral vascular disease *n* (%)	4 (9.5%)	0 (0.0%)	0.055
HT *n* (%)	23 (53.5%)	6 (14.0%)	<0.05
Anti-HT Treatment *n* (%)	19 (44.2%)	6 (14.0%)	<0.05
CAIs *n* (%)	17 (40.5%)	4 (9.3%)	<0.05
Diuretics *n* (%)	7 (16.7%)	1 (2.3%)	<0.05
Ca + Antagonists *n* (%)	2 (4.8%)	2 (4.7%)	1.000
Others *n* (%)	1 (2.4%)	3 (7.0%)	0.616
Statins *n* (%)	7 (16.7%)	2 (4.7%)	0.089
Fibrates *n* (%)	1 (2.5%)	0 (0.0%)	0.482
Antiplatelets *n*(%)	7 (17.5%)	0 (0.0%)	<0.05
Smoking habits			
*Non smoker*	23 (53.5%)	27 (62.8%)	0.487
*Current smoker*	4 (9.3%)	5 (11.6%)	
*Ex-smoker*	16 (37.2%)	11 (25.6%)	
*Patients*	43	43	

**Table 3:** Optical Coherence Tomography Angiography (OCTA) and structural Optical Coherence Tomography (OCT) characteristics (6 × 6 mm OCTA scan, 6 × 6 mm macular OCT Cube scan, and 6 × 6 mm Optic Nerve Head cube scan). Significant *p* values: Baseline-Month 1 (a); Baseline-Month 6 (b); Baseline-Month 12 (c); Baseline-Control (d); Month 1-Control (e); Month 6-Control (f); Month 12-Control (g). (FAZ = foveal avascular zone; RNFL: retinal nerve fiber layer; SD: standard deviation; SEM: standard error of the mean).

Characteristics, Mean (SD) (SEM)	Baseline	Month 1	Month 6	Month 12	Control	Significant *p*-Values
OCTA						
Vessel Density (mm^−1^)	16.70 (1.63)(0.26)	16.92 ^e^ (1.97)(0.34)	17.00 ^f^ (1.47)(0.27)	17.68 ^c^ (1.26)(0.23)	17.73 ^d^ (1.37)(0.21)	c,d,e,f
Perfusion Density (0–1)	0.406 (0.043)(0.007)	0.413 (0.051)(0.009)	0.415 ^f^ (0.039)(0.007)	0.433 ^c^ (0.035)(0.006)	0.434 ^d^ (0.035)(0.006)	c,d,f
FAZ Area (mm^2^)	0.232 (0.105)(0.018)	0.239 (0.119)(0.021)	0.247 (0.116)(0.022)	0.251 (0.115)(0.021)	0.274 (0.075)(0.014)	-
FAZ Perimeter (mm)	2.085 (0.574)(0.097)	2.014 (0.540)(0.097)	2.082 (0.540)(0.102)	2.077 (0.525)(0.094)	2.140 (0.333)(0.062)	-
FAZ Circularity	0.657 (0.110)(0.019)	0.704 ^a,e^ (0.082)(0.015)	0.684 ^f^ (0.092)(0.017)	0.698 ^g^ (0.087)(0.016)	0.743 ^d^ (0.068)(0.013)	a,d,e,f,g
STRUCTURAL OCT						
RNFL (μm)	92.47 (11.01)(1.68)	93.37 (9.88)(1.67)	93.74 (10.90)(1.96)	92.06 (9.77)(1.76)	95.73 (7.91)(1.24)	-
Central Retinal Thickness (μm)	260.47 (23.05)(3.52)	257.50 ^a^ (20.72)(3.45)	261.48 (22.16)(3.98)	261.81 (22.82)(4.10)	265.40 (22.95)(3.54)	a
Macular Volume	10.16 (0.38)(0.06)	10.09 ^e^ (0.30)(0.05)	10.22 (0.32)(0.06)	10.26 ^c^ (0.34)(0.06)	10.36 (0.54)(0.08)	c,e
Macular Thickness (μm)	282.36 (10.31)(1.59)	280.24 ^e^ (8.09)(1.41)	283.61 (8.95)(1.61)	284.90 ^c^ (9.50)(1.71)	287.50 (14.85)(2.29)	c,e
Ganglion-Cell Complex Thickness (μm)	82.02 (6.06)(0.95)	81.40 ^a^ (6.23)(1.05)	81.97 (5.85)(1.05)	81.65 (5.24)(0.94)	-	a
Eyes	43	36	31	32	43	

## Data Availability

Data will be made available upon request from the corresponding author. The data are not publicly available due to privacy concerns.

## Abbreviations

The following abbreviations are used in this manuscript (in alphabetical order):

AL	Axial length
AMT	Average macular thickness
BCVA	Best corrected visual acuity
BMI	Body mass index
CC	Choriocapillaris
CRT	Central retinal thickness
DCP	Deep capillary plexus
FAZ	Foveal avascular zone
FAZa	Foveal avascular zone area
FAZc	Foveal avascular zone circularity
FAZp	Foveal avascular zone perimeter
GCC	Ganglion cell complex
IQR	Interquartile range
MV	Macular volume
OCT	Optical Coherence Tomography
OCTA	Optical Coherence Tomography Angiography
PD	Perfusion density
RNFL	Retinal nerve fiber layer
SCP	Superficial capillary plexus
SD	Standard deviation
SE	Spherical equivalent
SVD	Small vessel disease
VD	Vessel density
